# Long-Term Weight Maintenance after a 17-Week Weight Loss Intervention with or without a One-Year Maintenance Program: A Randomized Controlled Trial

**DOI:** 10.1155/2015/651460

**Published:** 2015-03-30

**Authors:** Tuula Pekkarinen, Jarmo Kaukua, Pertti Mustajoki

**Affiliations:** ^1^Division of Endocrinology, Department of Medicine, Helsinki University Central Hospital, Peijas Hospital, Sairaalakatu 1, PL 900, 00029 HUS Vantaa, Finland; ^2^Sanofi Oy, PL 22, Huopalahdentie 24, 00350 Helsinki, Finland

## Abstract

*Background*. Weight lost by obese patients is almost always regained over time. Extended treatment may improve maintenance, but solid evidence is lacking. *Purpose*. We determined effectiveness of maintenance therapy after a weight loss program. *Methods*. Together 201 patients (mean age 47 years and BMI 42 kg/m^2^, 71% women) were randomly assigned to either a 17-week weight loss program followed by a one-year maintenance program or to a weight loss program without subsequent maintenance intervention. The weight loss program included behavior modification and a very-low-calorie diet, and maintenance program behavior modification. The primary outcome measure was percentage of patients with 5% or more weight loss at the end of maintenance (week 69) and one year later (week 121). Secondary outcomes were weight related changes in lifestyle and quality of life. *Results*. At week 69, 52% of the patients with and 44% of those without maintenance program had lost weight ≥5%, *P* = 0.40, and, at week 121, 33% and 34%, *P* = 0.77, respectively. At week 121 secondary outcomes did not differ between the groups among those successfully followed up. *Conclusions*. This one-year maintenance program was not effective in preventing weight regain in severely obese patients. *Trial Registration*. This trial is registered under clinicaltrials.gov Identifier: NCT00590655.

## 1. Introduction

Worldwide, obesity is one of the major public health problems with a recent trend towards more severe grade [1–3]. Numerous lifestyle interventions have well documented weight loss and beneficial end-treatment changes in obesity related modifiable cardiovascular risk factors but not in cardiovascular events [4–6]. After intervention, sustainability of new lifestyle and thereby lower weight has generally been humble in our obesogenic environment [7–9]. Therefore prevention of regain of the lost weight is a real challenge to patients and health care providers.

In the existing literature, there seems to be a consensus that extended treatment of obesity is associated with improved weight maintenance [9]. Much of this data comes from studies showing better weight outcome of interventions providing extended treatment or maintenance period after initial six months' weight loss. A recent meta-analysis showed an additional maintenance of 3.2 kg over 17.6 months with maintenance program compared with educational or no-contact control groups [10]. However, due to patient selection (maintenance phase offered only to those with good initial weight loss), these extended programs have shown efficacy in the most motivated patients [11, 12]. Among unselected patients on obesity treatment, the optimal dose of therapy needed to maintain new behavior and weight loss is less well known. Moreover, so far all maintenance studies have confirmed the problem of weight regain despite continuous care [11, 13, 14]. Also, a descriptive report of successful weight maintainers implies that high amount of exercise, restricted energy and fat intake, decreased consumption of fast food, and regular weighing are important factors for long term weight maintenance rather than extended treatment [15–17].

Whether compliance to new behaviors continues after successful maintenance treatment is another question. In 1988, Perri et al. [18] suspected that successful maintenance program postponed rather than prevented weight regain. In contrast, a recent systematic review of strategies for successful weight maintenance proposed that initial comprehensive weight loss program can carry over skills to follow up even without maintenance contacts [19].

This randomized controlled trial in severely obese patients was designed to compare weight loss maintenance after our usual care, a 17-week behavior modification program including a 10-week very-low-calorie diet (VLCD) (Group 1) with the same initial treatment followed by a one-year maintenance therapy with monthly meetings (Group 2). The primary hypothesis was that this maintenance phase would make the treatment more effective by increasing the number of patients who maintain improved lifestyle and meaningful weight loss at the end of treatment (week 69). Our special interest was long-term follow-up: we compared the outcome one year after the maintenance (week 121) to find out whether this program forestalls or postpones weight regain.

## 2. Methods

### 2.1. Study Design and Patients

This study was conducted at an outpatient obesity clinic, Peijas Hospital, Helsinki University Central Hospital, which provides treatment for residents in the surrounding communities with 250 000 inhabitants. Since 1995, primary and occupational health care has been able to refer a severely obese patient to specialist care if the patient is motivated to intensive intervention. For this study we invited all referred patients during the study inclusion time. The inclusion (BMI over 35 kg/m^2^, age 18–65 years, and stable weight three months) and exclusion criteria (contraindications to use VLCD, participating in the same treatment within five years, pregnancy, malignant disease, acute coronary event, current severe alcohol/narcotic abuse, or psychic problem/bulimia nervosa) were equal to the referral to our usual care. Contrary to usual care, visit to the endocrinologist was free (normal cost 30 €), group treatment was free (normal cost 120 €), and the patients received some VLCD for free (daily cost about 10 €).

At screening, patients visited an endocrinologist who was informed of the study, performed physical examination, obtained medical and weight history, and determined suitability for the study. Eligible patients confirmed their participation by giving informed written consent and filled in questionnaires. This study was approved by the Ethics Committee of Helsinki University Hospital.

### 2.2. Interventions

The interventionists, mainly staff of the hospital, included two nutritionists and six trained nurses who all had previous experience of weight loss groups. They were trained in both interventions and carried out the programs together with the research physicians. Four interventionists conducted both types of treatments. Consistency of programs was monitored in regular staff meetings.

*Seventeen-Week Weight Loss Program.* This program was similar in both treatments. The interventionists used a Finnish manual [20] based on the LEARN Programme for weight control [21]. Of the 17 sessions, group coaches guided 15 (1,5 hour each for groups of 13–15 patients), one was guided by a physiotherapist at gym or with Nordic walking and one by a physician discussing medical issues.

*VLCD*. The patients used VLCD (Nutrilett, Nutrifast, or Dietta Mini) as only diet during study weeks 2–11. These commercially available diets provide 52–58 g of protein, 52–64 g of carbohydrates, 8–13 g of fat and daily requirements of vitamins, trace elements, and minerals, and daily energy intake of 2200–2340 kJ. A moderate amount of vegetables was allowed.

During the first week the patients ate normal food and kept diary for self-monitoring. From the second week the VLCD started for ten weeks, followed by a two-week refeeding phase. The need to change previous energy intake and exercise habits after VLCD in order to maintain weight loss was emphasized. Each patient rather than therapist planned behavior modifications. Each session had one or two themes of behavior control, nutrition, or exercise with related homework. Themes included recording of eating and physical activity for self-monitoring, goal setting, regular weighing and regular meals, slowing down with eating, portion size, coping with overeating and eating impulses, importance of social support, lapses and relapse prevention, coping with risk situations, challenging negative thinking, problem solving, fat, fibre, sugar, and alcohol in diet, energy density of food, and energy expenditure. Increase in physical activity (like walking) and in lifestyle activity (using stairs and increasing number of steps) was repeatedly discussed, and participants were advised to buy and use a pedometer to monitor the amount of steps. Towards the end, focus was set on the importance of continuous self-monitoring.

*Maintenance Program.* The maintenance program was designed for this study. Each monthly session (1,5 hour) had one or two themes and related homework. The themes were monitoring eating, eating at regular times, control of eating impulses, fat and energy density in food, lifestyle activity and related energy expenditure, monitoring exercise and obstacles to increase exercise, importance of regular weighing, social support, body image changes, cooking/shopping together, lapses and relapses, problem solving, goal setting, and self-confidence. Two sessions were led by physiotherapist with Nordic walking or at gym.

### 2.3. Measurements

Weight was measured using a study-purchased digital scale with an accuracy of 0.1 kg (Soehnle model 7307, Soehnle-Waagen GmbH & Co, Murrhardt, Germany) with light clothing and no shoes at baseline, at each session and at weeks 69 and 121. If weight data was not obtained due to attrition, we calculated weight assuming that these patients had regained 0.3 kg per month after leaving the program. Body mass index (BMI) was calculated as weight in kilograms divided by the square of height in meters. At screening, weeks 17, 69, and 121, the participants filled in a self-report questionnaire concerning frequency of leisure-time exercise at least 30 min with some sweating or getting out of breath (daily/two or three times weekly/once weekly/two to three times monthly/a few times per year/unable to exercise due to physical limitation), eating three meals (always/almost always/rarely/hardly ever), choosing low fat food (every day/every week/now and then/do not pay attention to fat content of food), and weighing (every day/weekly/less often/no weighing).

At the same time points health-related quality of life (HRQOL) was assessed by the RAND 36-Item Health Survey 1.0 (RAND-36) [22]. The RAND-36 contains eight scales that include general health, physical functioning, and limitations on usual role-related activities due to physical health problems, bodily pain, energy and fatigue (vitality), limitations on usual role-related activities due to emotional or mental problems, social functioning, and emotional or mental health. Scores of all scales range from 0 to 100, with higher scores indicating better health or function.

### 2.4. Outcomes

The primary outcome measure was the difference between treatment groups in the percentage of patients with 5% weight loss or more from initial weight at weeks 69 and 121. The secondary outcome was differences in weight related behaviors and quality of life between the treatment groups at weeks 69 and 121.

### 2.5. Statistical Analysis

Several studies suggest that maintained weight loss of 5% is related to improved health status [9]. According to previous experience, one-third of patients in our usual care reach this goal at the two-year follow-up [23]. The sample size calculation indicated that if the new treatment results in 5% weight loss in two-third (67%) at two years, with 80% power and significance level of 0.05, at least 77 patients in each arm are needed. A total of 100 patients were chosen for each group. A physician who had no contact with the patients carried out randomization using a computer-generated table of random numbers with block size of four and allocated participants. Patients, interventionists, and investigators were not blinded.

Descriptive characteristics are reported as means with SD or as frequencies. The comparison for baseline characteristics between the study groups and comparison of dropouts versus nondropouts was performed with chi^2^ test or Mann-Whitney *U* test depending on the nature of variable of concern. The difference between the groups in the percentage of patients with 5% or more weight loss was tested by means of Fisher's exact test. General linear modelling, repeated measures procedure was used to compare weight, BMI, and weight loss percent between subjects (treatment groups) and within subjects, time with four (weight, BMI) or three (weight loss percent) levels. Partial correlation analysis was used to study correlates of weight loss. Baseline variables were tested as predictors of weight loss with multiple linear regression analyses, weight loss at week 69 or 121 as dependent and treatment group and each baseline variable, and treatment × each baseline variable as independent variables.

Leisure time exercise (at least twice weekly/less than twice weekly), eating meals daily (mostly three/less), choosing low fat food (daily/less often), and weighing (weekly/less often) were evaluated with chi^2^ test. Also, the change from baseline of these variables was examined similarly. The lifestyle change was analyzed after being classified to be improved or no change in previous beneficial behavior (leisure time exercise at least twice weekly, eating three meals mostly, choosing low fat food daily, weight weekly) or deteriorating to or continuing an unhealthy behavior (leisure time exercise less than twice weekly, not eating three meals mostly, not choosing low fat food daily, weighing less often than weekly). General linear modelling, repeated measures procedure was used to compare the RAND-36 scores between subjects (treatment groups) and within subjects (time with three levels). Missing questionnaires, 60 (30%) at week 17, 62 (31%) at week 69, and 102 (52%) at week 121, were not imputed.

Missing weight data, 51 (26%) at week 17, 34 (17%) at week 69, and 54 (27%) at week 121, were replaced using a conservative intention-to-treat method: we assumed these patients to have regained 0.3 kg per month after leaving the program [24]. Two men died and were not included in analysis after death. All reported* P* values are two-sided and *P* values < 0.05 are regarded as statistically significant. SPSS software (version 19) was used.

## 3. Results

### 3.1. Baseline Characteristics

**Figure 1:**
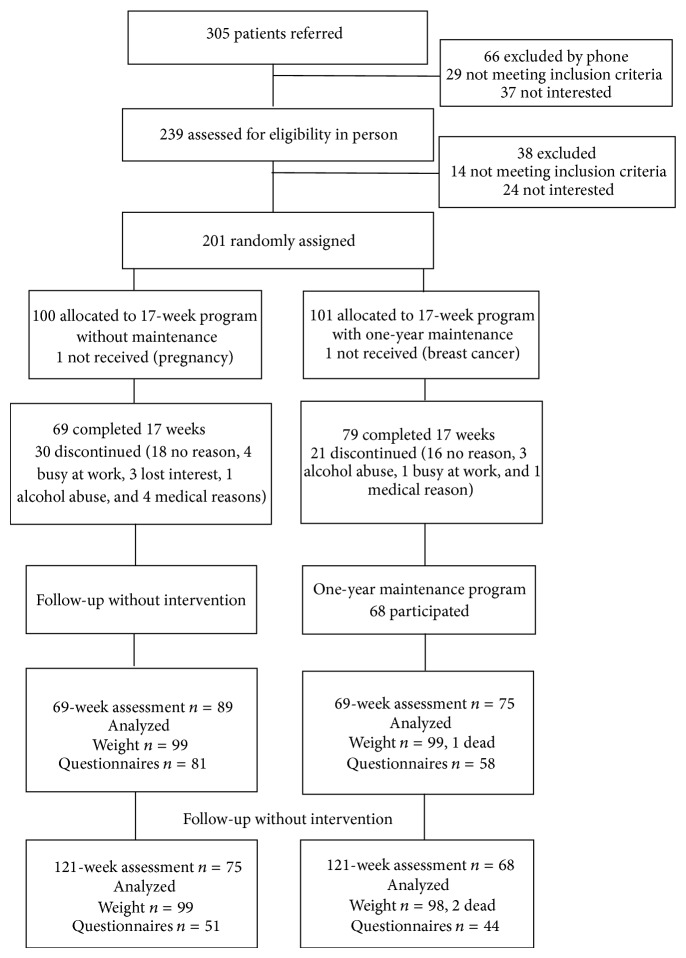
Flow of patients through the study.

Of the 305 individuals who were referred for obesity treatment and screened for eligibility, 239 (78%) visited endocrinologist and 201 (66%) were randomly assigned to two parallel groups: 100 to a 17-week weight loss program (Group 1) and 101 to a 17-week weight loss program followed by a one-year maintenance program with twelve monthly meetings (Group 2) (Figure 1). Fourteen groups (13 to 15 patients in each) started, four in 2002 (two groups of both types) and ten in 2003 (five groups of both types), and the last visit of the last group was in 2006. Two women found to be ineligible postrandomization were excluded from the analysis. The baseline characteristics were comparable except clinically diagnosed sleep apnoea which was more common in Group 2 (Table 1). The mean age was 47 years, mean weight was 119 kg, and most were women (*n* = 141, 71%), had BMI over 40 kg/m^2^ (*n* = 107, 54%), and were white (*n* = 198, 99%).

*Attrition during the 17-Week Intervention. *Together 148 patients completed the 17-week phase: 69 (70%) in Group 1 and 79 (79%) in Group 2, and 30 (30%) and 21 (21%) discontinued, respectively, *P* = 0.13. Participants who discontinued without medical reason were younger than those who continued, mean age 43.4 (SD 9.2) versus 48.6 (SD 10.3) years, respectively, *P* = 0.001. In Group 2, 11 did not start maintenance phase and 21 had previously dropped out.

### 3.2. Attendance at Scheduled Visits

Patients in Group 1 attended mean 12.9 (SD 4.4) and those in Group 2 13.2 (SD 4.1) of the 17 weekly sessions, *P* = 0.87. Two patients in Group 1 did not attend any session but were included in the analyses because they were aware of the group they were randomized in. The 68 subjects in Group 2 who actually participated the maintenance phase attended mean 6.4 (SD 3.3) of the 12 sessions.

### 3.3. Weight Loss

**Figure 2:**
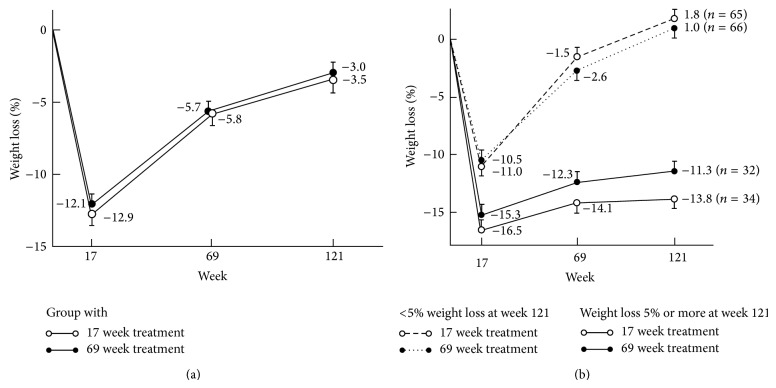
(a) Mean (SE) percentage change from baseline in weight over 121 weeks in the treatment groups. (b) Mean (SE) percentage change from baseline in weight over 121 weeks in the treatment groups according to the final outcome, weight loss ≥ 5% or < 5% at week 121.

Weight loss data are shown in Table 2 and Figures 2(a) and 2(b). Similar number of patients in both groups achieved 5% weight loss at all timepoints. There was a nonsignificant group × time interaction effect for change in weight, weight loss percent, and BMI. The mean weight loss of all patients at week 121 was 3.2% and 123 (62%) were below the baseline weight.  Figure 2(b) shows how weight change in successful participants (weight loss ≥ 5% at week 121) versus less successful (<5% weight loss at week 121) differed already at week 17 in both treatment groups.

### 3.4. Correlations and Predictors of Weight Loss

Partial correlation, which controlled for treatment, baseline weight, and therapist, showed that weight loss percent at week 17 correlated with weight loss percent at week 69 (*r* = 0.63, *P* < 0.0001) and at week 121 (*r* = 0.46, *P* < 0.0001) and the more the subjects attended sessions during the 17-week phase, the more the weight they had lost at week 17 (*r* = 0.68, *P* < 0.0001), at week 69 (*r* = 0.37, *P* < 0.0001), and at week 121 (*r* = 0.24, *P* = 0.001). In Group 2, the number of the attended maintenance sessions correlated with weight loss at week 69 (*r* = 0.45, *P* < 0.001) and at week 121 (*r* = 0.38, *P* < 0.001), adjusted for baseline weight and therapist.

There was no interaction with treatment and sex, age of the onset on obesity (child/adult), previously lost weight >10 kg (no, once, twice or more), basic education, employed (yes/no) on weight loss at week 69 or 121, but professional education × treatment interaction on weight loss at week 69 was found. In Group 1 those with vocational school or college and in Group 2 those with university education were most successful in weight loss, *P* = 0.01, and with adjustment for baseline weight *P* = 0.007, but no interaction was found at week 121 (*P* = 0.09). This suggests that the treatments had transient different effects in patients with different professional educations.

### 3.5. Life Style and Quality of Life

At baseline, weight related behaviors were similar between the groups (Table 1). At week 69 beneficial behaviors in exercise, use of low fat food, and weighing were more commonly reported in Group 2 compared with Group 1 (Table 3).

The baseline HRQOL scores in the RAND-36 between the study groups of all patients were similar (data not shown). Among those who successfully filled in the RAND-36 at all timepoints, the scores between the treatment groups were similar (Table 4). In this group of patients, the mean weight loss at week 121 was 5.5% (SD 8.8), nonsignificant between the treatments. Baseline scores in the RAND-36 of these patients versus those did not fill in the RAND-36 at all timepoints were not statistically different except for pain, where mean (SD) score was 63.5 (27.9) versus 72.5 (24.6), respectively, *P* = 0.02.

### 3.6. Adverse Events

Medical reasons to drop out during the 17-week phase (herpes zoster in eye, brain contusion, unstable angina pectoris, relapse in previously treated bulimia, and pneumonia) were not regarded as treatment related except the relapse in bulimia. From week 18 to week 121, 62 patients (equally distributed in both groups) were treated in secondary health care. Those who concomitantly dropped out of this study had pregnancies (*n* = 4), breast (*n* = 1) and prostate (*n* = 2) cancers, bariatric surgery (*n* = 2), Guillain-Barre polyradiculitis (*n* = 1), progression in diabetic nephropathy (*n* = 2), and stroke (*n* = 1). One patient died due to alcohol pancreatitis followed by stroke and another in an accident. Other reasons for secondary care but not attrition from the study were psychiatric therapy (*n* = 19), acute cholecystitis (*n* = 12), sleep apnoea diagnosed and treatment initiated (*n* = 8), hospitalization due to heart failure (*n* = 1), unstable angina pectoris (*n* = 1), brain aneurysm operation (*n* = 1), knee prosthesis (*n* = 2), low back pain (*n* = 1), and hepatitis C (*n* = 1).

### 3.7. Costs of the Maintenance Program

The treatment with maintenance program was more expensive than our usual care. We calculate four hours of work (2 for preparing, 1.5 for the session, and 0.5 for administration) for the interventionist to conduct one session. Thus, maintenance phase per each group with 15 patients was 48 hours extra work and 3200 € extra cost.

## 4. Discussion

Compared with our usual care, the therapy with a one-year maintenance program after a 17-week weight loss phase did not prevent or delay weight regain in severely obese patients in this study. Equal number of patients, one-third, sustained clinically significant 5% weight loss over two years. We confirmed the results of several earlier studies showing that patients who respond well during the early phase of the weight loss program had larger weight loss on long-term [14, 25–27] and in this study regardless of the length of the treatment. In accordance with previous studies, we found higher attrition among young patients [27, 28].

Consistent with our finding, one meta-analysis of observational studies and another of randomized controlled trials did not find correlation with the length of obesity treatment and weight loss [29, 30]. Our results differ from the reports of better weight maintenance seen with extended care [10]. The fact that only successful weight losers were accepted to those maintenance studies may explain the discrepancies in results. In our study, weight loss success in Group 2 correlated with higher attendance in maintenance sessions. Thus, the motivated and thereby successful weight maintainers appeared to take advantage of the long treatment by attending the provided sessions, as previously shown in the Look AHEAD study [14]. However, in our study the motivated patients in Group 1 successfully adopted maintenance strategies during the 17-week multidisciplinary treatment showing that the necessary tools for maintenance can be provided during a relatively short program. Therefore, given the cost and time involved in extended therapy and the increasing burden of obesity in health care, this maintenance program is hard to justify in our daily practice.

Weight loss after VLCD was challenging to sustain in our study like in many previous studies. One of the main causes is poor compliance with new behaviors [31]. Despite similar weight results at week 69, Group 2 reported beneficial weight related behaviors more often compared with Group 1. This may be due to desire to please the study personally but also due to our insensitive tool to measure and grade weight related behaviors. Regaining weight after VLCD has also been related to adaptive thermogenesis, reduced energy expenditure, and changes in appetite mediating hormones [32, 33]. Perhaps partly because of these factors, some improvement in maintenance after VLCD has been reported with antiobesity drugs, meal replacements, and high-protein diets [27]. In addition, in those studies treatment effect may be overestimated by use of completers' weight data or last observation carried forward analysis with a relatively short study duration compared with our study [27]. We analyzed weight with a conservative and probably more accurate method by adding 0.3 kg per month after withdrawal.

Besides these dissimilarities in data analyzing methods, the effect of recruitment type may explain discrepancies in weight maintenance studies. An observational Swedish cohort study of self-selected, self-paying motivated adults reported mean 11% weight loss at one year [34]. Interestingly, they reported an increased risk for attrition in participants with depression medication. In our study primary care physicians referred the patients, most of whom had previously been unsuccessful in maintaining large weight losses. Self-efficacy and other psychological factors which probably mediate weight outcome on long term may be different in these patient groups. Another self-referral study randomized severely obese patients into an intensive treatment (26 visits after weight loss phase) and a usual care and reported mean loss of 4.9% and 0.2%, respectively, at month 24 [35]. Our maintenance program with 12 visits was less intensive and this may be one reason for less successful outcome. However, in the Look AHEAD study with highly intensive lifestyle intervention combining diet, exercise, and behavioral therapy weight regain during maintenance was inevitable [14]. Moreover, similar weight regain and poor long term weight maintenance has been reported both after rapid (VLCD) and slow weight loss despite additional counseling for those who started to regain weight [36]. Clearly, future studies are needed to exam weight maintenance.

We report comparable HRQOL scores between the treatment groups among those who filled in the RAND-36 at all timepoints and sustained weight loss similarly. In quality of life research, a concept of minimal clinically important difference (MCID) has been proposed to refer to the smallest difference in score that is considered to be worthwhile or important. The MCID for the RAND-36 has been suggested to be three to five points [37, 38]. Within the groups, weight loss and partial regain over 121 weeks were associated with durable improvement of seven to eight points in the scores of physical functioning. Thus, the long term improvement in physical functioning in both groups of this study is probably meaningful and has been reported previously [23, 39]. Several other domains improved transiently showing a mirror image between HRQOL changes and weight loss and regain as previously reported [40]. However, deteriorations below baseline in social functioning and mental health scores in Group 1 and in emotional functioning score in Group 2 were unexpected and need to be studied further.

Our study also had limitations. First, the low number of questionnaires filled in at weeks 69 and 121 and use of a self-report questionnaire rather than objective measures of weight related behaviors are inevitable causes of error in estimating true changes in behaviors and quality of life. Second, it is extremely difficult to blind staff and participants in a lifestyle intervention, and nonblinding is one weakness of our study. Being aware of the aim of our study, the participants in Group 1 may have specially focused in the prevention of weight regain on their own. However, the study by Kaukua et al. [23] with identical 17-week program resulted in similar outcome as this study: one-third had 5% weight loss or more two years later. Third, these results concern severely obese patients and may not be applicable to other patient groups.

Despite these limitations, this pragmatic study has several strengths including high number of patients, long term follow-up and real-life clinical setting which makes this outcome informative regarding what happens in daily practice.

We conclude that among severely obese patients this one year maintenance program after a multidisciplinary 17-week weight loss therapy with VLCD had no major effect on preventing weight regain at the end of the maintenance or one year later.

## Figures and Tables

**Table 1:** Baseline demographic and clinical characteristics of the patients by treatment group, data are means (SD) or numbers (%).

	Group 1 *N* = 99	Group 2 *N* = 100

Age (years)	47.3 (10.5)	47.4 (10.1)
Weight (kg)	120.6 (23.5)	117.8 (22.0)
Height (m)	1.69 (0.10)	1.69 (0.08)
BMI (kg/m^2^)	42.1 (5.7)	41.4 (6.4)
Female	71 (72%)	71 (71%)
Married/living together	60 (72%)	56 (78%)
Childhood obesity	33 (33%)	39 (39%)
Earlier lost >10 kg		
Never	22 (22%)	28 (28%)
Once	49 (49%)	46 (46%)
Twice or more	28 (28%)	26 (26%)
Smoking	32 (32%)	26 (26%)
Basic education		
Primary school	30 (31%)	25 (25%)
Comprehensive school	43 (44%)	55 (55%)
High school	25 (26%)	20 (20%)
Professional education		
No	12 (13%)	12 (12%)
Vocational courses	30 (32%)	31 (32%)
Vocational school	17 (18%)	21 (22%)
College	29 (31%)	24 (25%)
University	7 (7%)	9 (9%)
Employed	64 (65%)	70 (70%)
Diabetes medication	11 (11%)	22 (22%)
Hypertension medication	45 (45%)	49 (49%)
Lipid medication	12 (12%)	14 (14%)
Asthma medication	15 (15%)	12 (12%)
Psychiatric diagnosis	16 (16%)	14 (14%)
Sleep apnoea diagnosed	6 (6%)	18 (18%)
Leisure time exercise twice weekly or more	27 (28%)	37 (38%)
Eating three meals daily	53 (54%)	56 (57%)
Choosing low fat food daily	44 (45%)	46 (47%)
Weighing weekly	32 (33%)	41 (42%)

**Table 2:** Number (%) of patients with 5% or more weight loss, mean weight change, mean weight (SD), and BMI (SD) by treatment condition.

Outcome variable	Group 1 17-week program	Group 2 17-week + maintenance	*P*
Weight loss ≥5%			
Week 17	89 (90)	89 (89)	1.00^*^
Week 69	44 (44)	51 (52)	0.40^*^
Week 121	34 (34)	32 (33)	0.77^*^
Weight change % (95% CI)			
0 to week 17	−12.9 (−11.7 to −14.1)	−12.1 (−10.9 to −13.3)	
0 to week 69	−5.8 (−4.4 to −7.4)	−5.7 (−4.1 to −7.1)	0.71^**^
0 to week 121	−3.5 (−1.8 to −5.2)	−2.9 (−1.3 to −4.6)	
Weight (SD)			
Baseline	120.6 (23.5)	117.8 (22.0)	0.53^**^
Week 17	105.0 (22.0)	103.8 (22.3)	
Week 69	113.8 (25.9)	111.3 (23.0)	
Week 121	116.6 (27.2)	114.4 (23.1)	
BMI (SD, kg/m^2^)			
Baseline	42.1 (5.7)	41.4 (6.4)	0.43^**^
Week 17	36.7 (5.9)	36.4 (6.7)	
Week 69	39.7 (6.9)	39.0 (6.9)	
Week 121	40.7 (7.4)	40.1 (6.9)	

^∗^By Fisher's exact test, ^∗∗^ *P* value for test of treatment effect (group × time interaction) in general linear modelling, repeated measure procedure.

**Table 3:** Change in self-reported weight related behaviours of those who successfully filled in questionnaires in Group 1 (17-week program) and Group 2 (17-week program with maintenance).

	Number of answers	Increased to or maintained good behaviour		*P* between Group 1 and Group 2
	Group 1/Group 2	Group 1 *N* (%)	Group 2 *N* (%)	
Exercise twice weekly or more				
0–17 weeks	65/73	51 (78.5)	48 (66)	0.13
0–69 weeks	80/59	42 (52.5)	42 (71)	0.035
0–121 weeks	48/44	29 (60)	24 (54.5)	0.07
Eating 3 meals daily				
0–17 weeks	66/73	52 (79)	52 (71)	0.33
0–69 weeks	83/59	48 (58)	33 (56)	0.38
0–121 weeks	51/43	33 (65)	26 (60.5)	0.83
Choosing low fat food daily				
0–17 weeks	66/73	64 (97)	69 (94.5)	0.58
0–69 weeks	83/59	57 (69)	53 (90)	0.004
0–121 weeks	51/43	46 (90)	37 (86)	0.75
Weighing weekly				
0–17 weeks	66/73	61 (92)	72 (99)	0.10
0–69 weeks	82/59	50 (61)	46 (78)	0.04
0–121 weeks	51/43	28 (55)	27 (63)	0.53

**Table 4:** Mean quality of life scores (SD) in Group 1 (17-week program) and Group 2 (17-week program with one-year maintenance) during the trial.

Quality of life measure	Group 1		Group 2		*P*
	*N*		*N*		Group × time effect
General health					
Baseline		50.6 (17.0)		54.7 (15.6)	0.27
Week 69	50	54.1 (21.9)	38	64.3 (17.8)	
Week 121		50.9 (21.8)		57.4 (21.2)	
Physical functioning					
Baseline		61.4 (23.8)		69.9 (18.6)	0.82
Week 69	49	73.2 (25.0)	38	83.3 (16.9)	
Week 121		70.6 (24.9)		78.4 (21.1)	
Physical role functioning					
Baseline		53.1 (44.0)		68.4 (39.3)	0.94
Week 69	48	63.4 (43.1)	38	77.6 (35.3)	
Week 121		55.0 (42.1)		72.1 (40.6)	
Bodily pain					
Baseline		61.0 (28.2)		69.4 (25.8)	0.99
Week 69	49	65.2 (28.8)	38	74.3 (26.2)	
Week 121		59.5 (28.4)		68.4 (29.8)	
Energy					
Baseline		58.2 (20.8)		65.9 (19.0)	0.54
Week 69	48	56.8 (23.3)	38	69.3 (20.4)	
Week 121		56.0 (23.1)		66.1 (20.2)	
Social functioning					
Baseline		80.4 (24.7)		84.2 (17.4)	0.09
Week 69	49	74.7 (26.7)	38	86.5 (21.6)	
Week 121		71.9 (27.4)		85.9 (19.3)	
Emotional role functioning					
Baseline		63.5 (42.8)		84.7 (29.0)	0.76
Week 69	47	61.7 (45.0)	37	87.4 (27.6)	
Week 121		61.7 (42.8)		81.1 (32.9)	
Mental health					
Baseline		75.7 (16.8)		79.2 (17.0)	0.22
Week 69	47	71.1 (22.2)	38	81.2 (16.9)	
Week 121		71.1 (21.2)		77.9 (19.4)	
